# Long Non-Coding RNA *Malat1* Regulates Angiogenesis in Hindlimb Ischemia

**DOI:** 10.3390/ijms19061723

**Published:** 2018-06-11

**Authors:** Xuejing Zhang, Xuelian Tang, Milton H. Hamblin, Ke-Jie Yin

**Affiliations:** 1 Pittsburgh Institute of Brain Disorders & Recovery, Department of Neurology, University of Pittsburgh School of Medicine, Pittsburgh, PA 15213, USA; xuz20@pitt.edu (X.Z.); xut5@pitt.edu (X.T.); 2 Department of Pharmacology, Tulane University School of Medicine, 1430 Tulane Avenue SL83, New Orleans, LA 70112, USA; mhambli@tulane.edu

**Keywords:** long non-coding RNA, *Malat1*, angiogenesis, vascular endothelial cells, VEGFR2, hindlimb ischemia

## Abstract

Angiogenesis is a complex process that depends on the delicate regulation of gene expression. Dysregulation of transcription during angiogenesis often leads to various human diseases. Emerging evidence has recently begun to show that long non-coding RNAs (lncRNAs) may mediate angiogenesis in both physiological and pathological conditions; concurrently, underlying molecular mechanisms are largely unexplored. Previously, our lab identified metastasis associates lung adenocarcinoma transcript 1 (*Malat1*) as an oxygen-glucose deprivation (OGD)-responsive endothelial lncRNA. Here we reported that genetic deficiency of *Malat1* leads to reduced blood vessel formation and local blood flow perfusion in mouse hind limbs at one to four weeks after hindlimb ischemia. *Malat1* and vascular endothelial growth factor receptor 2 (*VEGFR2*) levels were found to be increased in both cultured mouse primary skeletal muscle microvascular endothelial cells (SMMECs) after 16 h OGD followed by 24 h reperfusion and in mouse gastrocnemius muscle that underwent hindlimb ischemia followed by 28 days of reperfusion. Moreover, *Malat1* silencing by locked nucleic acid (LNA)-GapmeRs significantly reduced tube formation, cell migration, and cell proliferation in SMMEC cultures. Mechanistically, RNA subcellular isolation and RNA-immunoprecipitation experiments demonstrate that *Malat1* directly targets VEGFR2 to facilitate angiogenesis. The results suggest that *Malat1* regulates cell-autonomous angiogenesis through direct regulation of VEGFR2.

## 1. Introduction

Angiogenesis, mainly stimulated by hypoxia in ischemic tissue, refers to the growth of new capillary blood vessels from the pre-existing vasculature. The formation process of new angiogenic vessels involve the proliferation, migration, and differentiation of blood vascular endothelial cells (ECs) [1]. Angiogenesis plays pivotal physiological functions in embryonic development, wound repair, tissue engineering, and placental development [1]. However, when dysregulated, the formation of new blood vessels may contribute to numerous oncogenic, ischemic, infectious, and inflammatory diseases [1,2,3]. At present, both anti- and pro-angiogenic treatments have therapeutic implications in human diseases [4]. For example, inhibition of angiogenesis can be beneficial in disorders such as cancer, arthritis, psoriasis, ophthalmic conditions, and hemangioma, whereas stimulation of angiogenesis can be beneficial in disorders such as ischemic stroke, ischemic heart disease, placental insufficiency, and other ischemia-associated diseases [4,5,6].

Importantly, the angiogenic response is strictly controlled by a coordinated regulation of both pro-angiogenic and anti-angiogenic molecules [7]. Dysregulation of transcription during this process favors pathological angiogenesis and accumulates endogenous pro-angiogenic molecules including growth factors, matrix metalloproteinases (MMPs), cytokines, and integrins [8,9]. Specifically, the major signaling molecule for angiogenesis is vascular endothelial growth factor (VEGF) which is commonly found to be upregulated in pathological conditions such as cancer and ischemic diseases [10,11]. VEGF promotes endothelial cell survival, proliferation, migration, and differentiation. Hypoxia induces VEGF expression via hypoxia inducible factor 1α (HIF-1α), which, in turn, activates VEGF receptor 2 (VEGFR2) to stimulate tip cell migration from arteries to initiate angiogenesis [12]. VEGFR2 is known as the major mediator of angiogenic signaling in blood vascular endothelial cells and is required for angiogenesis [13].

LncRNAs are RNA transcripts with no protein-coding ability that are more than 200 nucleotides in length [14]. Advances in whole genome transcriptomic analysis have revealed that large number of lncRNAs are involved in various biological activities, such as cell proliferation [15], muscle differentiation [16], organogenesis [17], chromatin remodeling [18], and genomic imprinting [19]. Previous studies have elucidated the importance of small non-coding RNAs, such as microRNA, in the regulation of angiogenesis [20,21]. However, only a few lncRNAs have been shown to contribute to vascular disease and endothelial cell integrity. Recent evidence indicates that lncRNA can regulate different processes involved in angiogenesis through direct or indirect mechanisms, mainly by regulating angiogenic molecules such as VEGF [22,23,24,25]. For example, lncRNA MEG3 knockout mouse presented increased expression of VEGF pathway genes and increased cortical microvessel density by qPCR analysis and immunohistological staining [22]. Another well-studied lncRNA *HOTAIR* was reported to promote angiogenesis through activating VEGFA transcription in both in vitro and in vivo nasopharyngeal carcinoma studies [23]. LncRNA *Punisher* has been identified in endothelial cell differentiation and angiogenesis by RNA-sequencing study, and inhibition of *Punisher* resulted in defective branching and compromised vessel formation [24]. In a recent study, silencing of lncRNA *MANTIS* inhibited angiogenic sprouting of HUVECs and the alignment of endothelial cells in response to shear stress [25]. Of note, the effects of these lncRNAs on endothelial cell biology and angiogenesis have been identified in both cultured endothelial cells and in ischemia-induced angiogenesis.

*Malat1* is one of the most extensively studied lncRNAs that is associated with human diseases [26]. *Malat1* gene is located within human chromosome 11q13 and mouse chromosome 19qA [27]. It is abundantly expressed at a level comparable with or even higher than many protein-coding genes, including β-actin or glyceraldehyde 3-phosphate dehydrogenase (*GAPDH*) [28]. Since its discovery, accumulating data from various basic and clinical studies have provided insights into its biogenesis, cellular, and molecular functions. Previously, we and others have demonstrated that *Malat1* can be significantly upregulated by oxygen-glucose deprivation (OGD), hypoxia, and hyperglycemia in endothelial cells [29,30,31]. Additionally, recent studies have shown that mice with genetic deletion of *Malat1* presented reduced retinal vascular growth and endothelial proliferation [32]. Moreover, pharmacological inhibition of *Malat1* using locked nucleic acid (LNA)-GapmeRs (single-stranded oligonucleotides designed to specifically silence *Malat1*) significantly decreased blood flow recovery and capillary density after hindlimb ischemia by impairing the expression of several cell cycle regulators [32]. However, whether *Malat1* can regulate cell autonomous angiogenesis in mouse vascular endothelial cells, especially during in vivo settings, remains unexplored.

In the present study, we utilized the *Malat1*-deficient mice model to explore the effects and molecular mechanisms of *Malat1* on hindlimb ischemia-induced angiogenesis. Laser Doppler imaging and capillary density analysis indicated that genetic deletion of *Malat1* significantly inhibited blood flow recovery and capillary density in gastrocnemius muscle tissue after hind limb ischemia. Furthermore, in vitro study confirmed that silencing of *Malat1* by LNA-GapmeRs inhibited proliferation, wound closure, and tube formation of mouse primary skeletal muscle microvascular endothelial cells (SMMECs). The mechanism of action for mediating these effects involved *Malat1* direct binding and regulating of VEGFR2. Collectively, these results suggest that *Malat1* may play critical vascular regulatory roles in promoting angiogenesis.

## 2. Results

### 2.1. Reduction of Local Blood Flow and Vascular Density in Malat1-Deficient Mice after Hindlimb Ischemia

**Figure 1:**
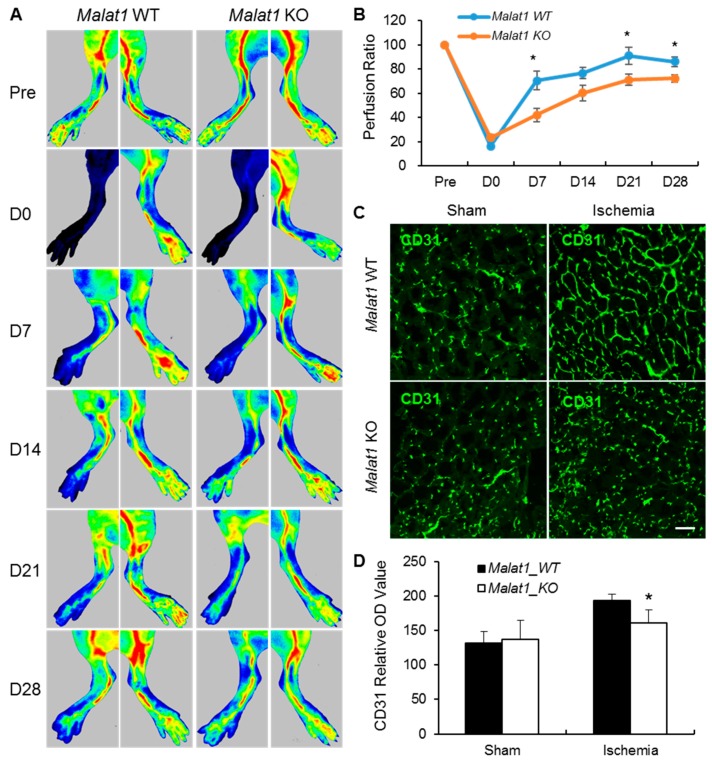
Effects of *Malat1* genetic deficiency on local blood flow recovery after mouse hindlimb ischemia. (**A**,**B**) *Malat1* knockout (*Malat1* KO) and littermate control (*Malat1* WT) mice were subjected to right femoral artery ligation and subsequently monitored by Laser Speckle imaging at days 0, 7, 14, 21, and 28 after hindlimb ischemia. The laser speckle pattern is visualized as a color-coded blood flow velocity with low flow = **blue**; high flow = **red**. Representative images are shown in panel (**A**) and quantification of blood flow recovery is shown in panel (**B**) (*n* = 8 per group). (**C**) Representative immunofluorescent images showing CD31-positive capillaries in transverse sections of non-ischemic (Sham-operated) and ischemic hindlimb gastrocnemius muscles from *Malat1* WT and KO mice (*n* = 5). Green color indicates CD31-positive staining; and (**D**) Quantitative analysis of CD31-positive capillaries in panel (**C**). In comparison with *Malat1* WT controls, genetic deficiency of *Malat1* significantly delays blood flow recovery and reduces vascular density in hindlimbs following ischemic insults. Data are expressed as mean ± SEM. * *p* < 0.05 vs. *Malat1* WT + ischemia group. Scale bar represents 50 µm.

To define the function of *Malat1* in vivo, we employed a silencing strategy using *Malat1* knockout (KO) mice on a C57BL/6J background [28]. *Malat1* KO and littermate control (WT) mice were subjected to hindlimb ischemia by femoral artery ligation. Local blood flow and capillary density in response to ischemic injury were determined by a laser speckle imager (Perimed PeriCam PSI HR, Stockholm, Sweden) (LSI) and CD31 immunofluorescence staining. In comparison with *Malat1* WT mice, recovery of blood flow in ischemic limbs was significantly delayed in *Malat1* KO mice at days 7, 21, and 28 after ischemia (Figure 1A,B). Representative CD31 staining images of the sham and ischemic hindlimb muscle in both *Malat1* WT and *Malat1* KO groups at postoperative day 28 are shown in Figure 1C. The number of capillaries in ischemic hindlimbs was dramatically reduced in *Malat1* KO mice compared with *Malat1* WT controls (Figure 1D), suggesting that *Malat1* may play functional roles in pro-angiogenesis after surgical induction of hindlimb ischemia. Of note, genetic deletion of *Malat1* in mice had no effects on local blood flow and capillary density under non-ischemic conditions (Figure 1A–D).

### 2.2. Genetic Deficiency of Malat1 Decreased Pro-Angiogenic VEGFR2 Expression after Hindlimb Ischemia

**Figure 2:**
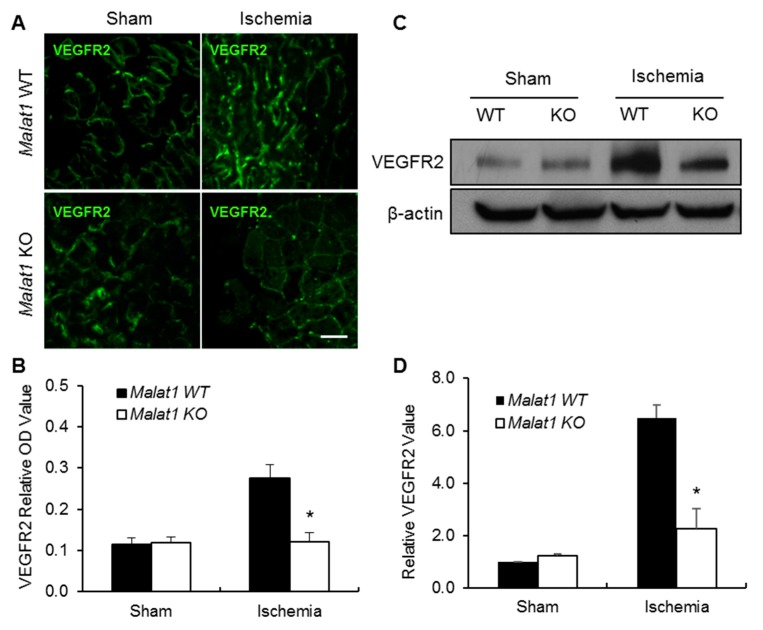
Genetic deletion of *Malat1* gene attenuates VEGFR2 expression in ischemic hindlimb gastrocnemius muscles. (**A**) Representative immunofluorescent images showing VEGFR2-positive vessel-like structures in transverse sections of sham and ischemic hindlimb gastrocnemius muscles from *Malat1* WT and *Malat1* KO mice (*n* = 5); (**B**) Quantitative analysis of VEGFR2-positive staining in panel (**A**); (**C**) The protein levels of VEGFR2 were determined by Western blotting, with β-actin as the loading control; and (**D**) Quantitative analysis of VEGFR2 expression in sham and ischemic hindlimb gastrocnemius muscles from *Malat1* WT and *Malat1* KO mice. Experiments were repeated three times and representative blots are displayed. Data are expressed as mean ± SEM. * *p* < 0.05 vs. *Malat1* WT + ischemia group. Scale bar represents 50 µm.

It has been largely accepted that VEGFR2 is the major mediator of angiogenic signaling in vascular endothelial cells and is required for angiogenesis [13]. Therefore, we examined VEGFR2 expression in ischemic hindlimb muscle at postoperative day 28 by immunofluorescence staining. The average VEGFR2 positive vessel-like structures were significantly decreased in ischemic hindlimbs of *Malat1* KO mice compared with *Malat1* WT controls (Figure 2A,B). The decreased VEGFR2 expression in *Malat1* KO ischemic hindlimb muscle was also confirmed at the protein level by Western blotting (Figure 2C,D).

### 2.3. Silencing of Malat1 Reduces Pro-Angiogenic VEGFR2 Expression in Mouse SMMEC Cultures

**Figure 3:**
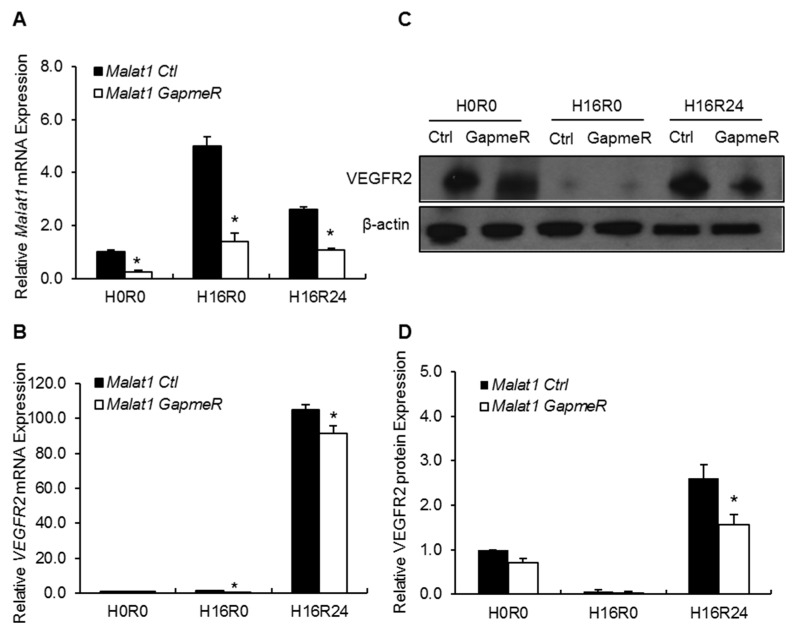
The effect of *Malat1* silencing on VEGFR2 expression in mouse SMMECs after OGD and reperfusion. LNA-GapmeRs targeting *Malat1* gene (*Malat1* GapmeR) or scrambled control GapmeRs (*Malat1* Ctrl) were transfected at 50 nM. (**A**) *Malat1* and (**B**) VEGFR2 expression levels were examined by qPCR and normalized to Cyclophilin (*n* = 3). qPCR data showed significantly reduced *Malat1* levels in *Malat1* GapmeR group in the absence (H0R0) or presence of 16 h OGD exposure (H16R0) and 16 h OGD followed by 24 h reperfusion (H16R24); (**C**) The protein level of VEGFR2 was determined by Western blotting, with β-actin as the loading control; and (**D**) Quantitative analysis of VEGFR2 expression in *Malat1* control and *Malat1* GapmeR transfected SMMEC cells in H0R0, H16R0, and H16R24 groups. Experiments were repeated three times and representative blots are displayed. Data are expressed as mean ± SEM. * *p* < 0.05 vs. *Malat1* Ctrl group.

To further uncover functional roles and molecular mechanisms of *Malat1* on vascular endothelium in response to ischemic stimuli, mouse primary SMMECs were subjected to OGD for 16 h, followed by 24 h reperfusion. We also employed single-stranded oligonucleotides *Malat1* GapmeRs [33] as a genetic approach to achieve *Malat1* silencing in mouse SMMEC cultures. *Malat1* expression can be induced by ischemic stimuli [30,31,32,34]. As shown in Figure 3A, *Malat1* displayed a five-fold increased expression after 16 h OGD exposure and kept at 2.5-fold increased expression after 24 h reperfusion in SMMEC cultures (Figure 3A). The basal and OGD-induced *Malat1* levels were significantly decreased by transfection with LNA-*Malat1* GapmeR versus transfection with the *Malat1* control in SMMEC cultures (Figure 3A). Treatment with *Malat1* GapmeR also significantly decreased OGD-induced *VEGFR2* expression at mRNA level when compared to the *Malat1* control group (Figure 3B). Moreover, OGD-induced VEGFR2 level was significantly decreased by *Malat1* GapmeR treatment at protein level by Western blotting (Figure 3C,D). The results suggest *VEGFR2* as a downstream target of *Malat1* in mouse vascular endothelial cells.

### 2.4. Malat1 Mediates SMMEC Cell Migration and Proliferation

**Figure 4:**
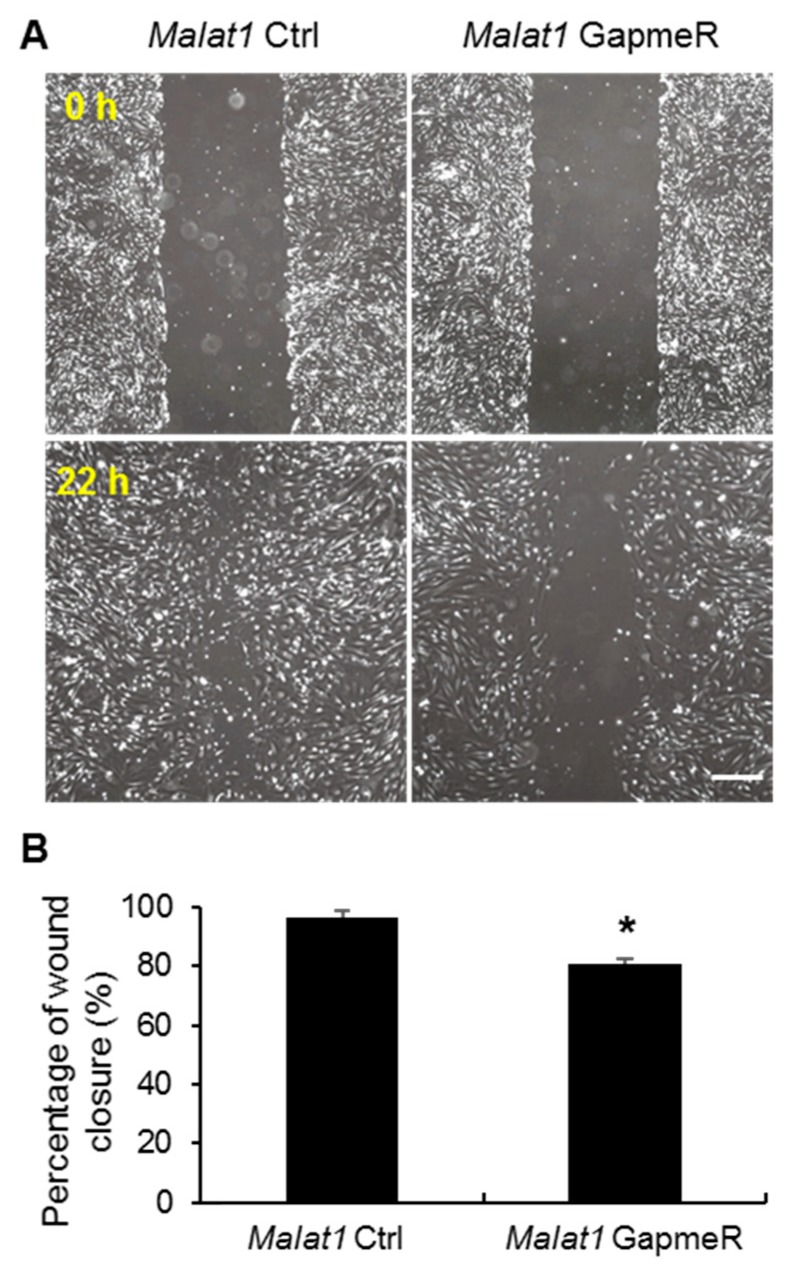
*Malat1* silencing reduces cell migration in SMMECs. (**A**) Representative photomicrographs of in vitro wound healing assays. SMMCEs were transfected with either *Malat1* control (*Malat1* Ctrl) or *Malat1* GapmeR for 48 h in 6-multiwell plates. Wounds were produced in SMMECs by a **yellow** pipet tip, and photomicrographs were taken as time 0 h. After an additional 22 h of incubation, photomicrographs were taken again; and (**B**) Cellular migration was calculated by measuring the area occupied by the migrated cells. In comparison to *Malat1* control (*Malat1* Ctrl) group, *Malat1* silencing by GapmeR treatment in SMMECs significantly damped cell migration. Results are mean ± SEM of three independent experiments. * *p* < 0.05 vs. *Malat1* Ctrl group. Scale bar represents 100 µm.

**Figure 5:**
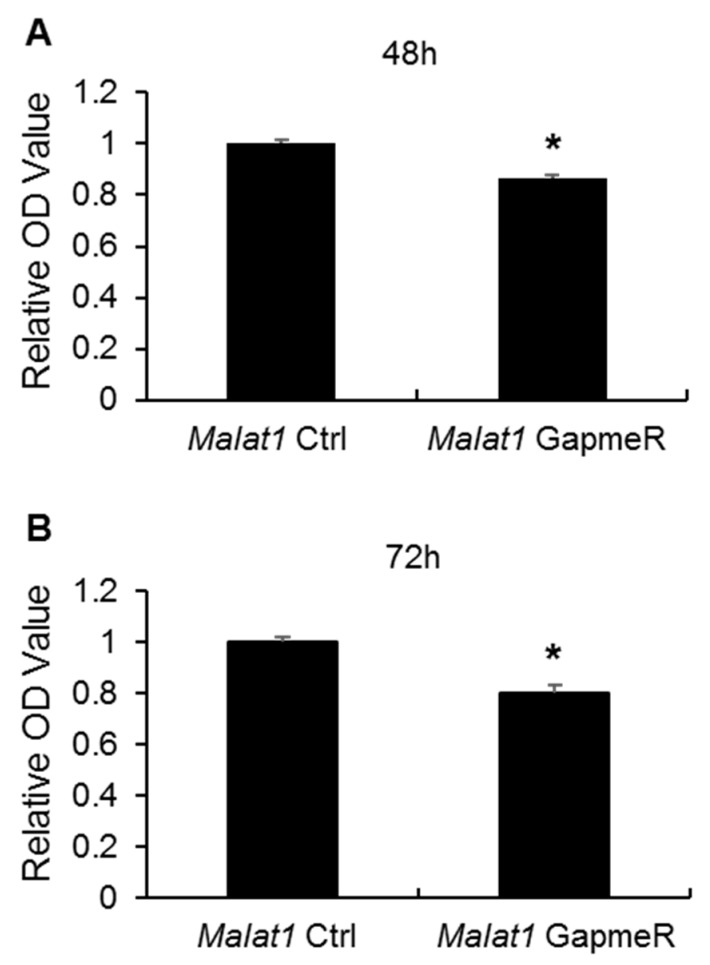
Effects of *Malat1* silencing on cell proliferation. SMMECs were transfected with either *Malat1* control (*Malat1* Ctrl) or *Malat1* GapmeR for (**A**) 48 h and (**B**) 72 h. DNA synthesis was measured by BrdU incorporation assays as described in the Methods. The results are expressed as mean ± SEM of three independent experiments. * *p* < 0.05 vs. *Malat1* Ctrl group.

It has been accepted that endothelial cell migration is a crucial step in angiogenesis and cell invasion [35]. To further assess the regulatory effect of *Malat1* to angiogenesis, in vitro scratch assays were performed in mouse SMMEC cultures following *Malat1* control/GapmeR treatment. As shown in Figure 4, silencing of *Malat1* in SMMECs markedly reduced cell migration in comparison with the *Malat1* control transfected group (Figure 4A,B). In addition, angiogenesis requires the proliferation of vascular endothelial cells; accordingly, we also tested whether silencing of *Malat1* affects SMMEC proliferation by using Bromodeoxyuridine (BrdU) incorporation assays. We found that *Malat1* silencing in SMMECs effectively inhibited proliferation by reducing DNA synthesis 48 h and 72 h after GapmeR treatment (Figure 5A,B). Thus, our results suggest that *Malat1* plays pro-angiogenic roles in SMMECs through modulating cell migration and proliferation.

### 2.5. Malat1 Regulates SMMEC Tube Formation

**Figure 6:**
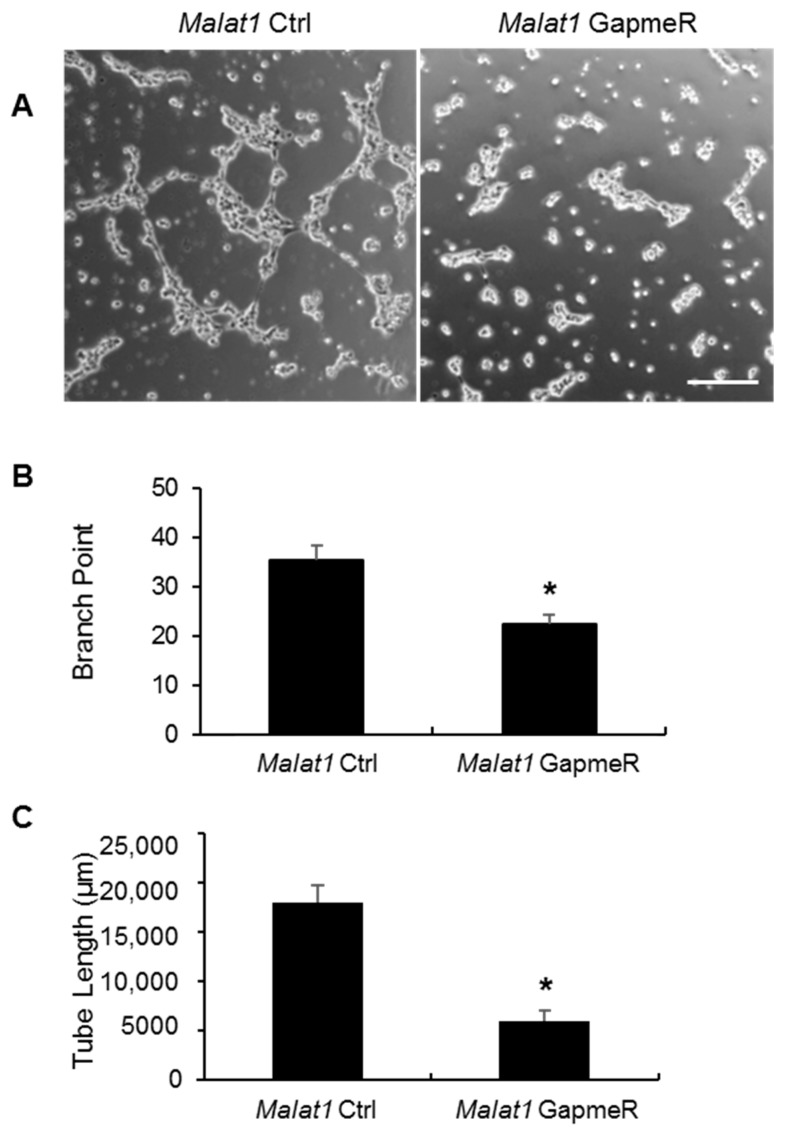
Effects of *Malat1* silencing on tube formation. SMMECs were transfected with either *Malat1* control (*Malat1* Ctrl) or *Malat1* GapmeR for 48 h and then treated with 1 ng/µL VEGF for 24 h. The cells were then re-seeded in BD matrigel matrix-coated 24-multiwell plates (1 × 10^5^ cells/well) in complete mouse endothelial cell medium (Cell Biologics, Chicago, IL, USA) for 6 h. (**A**) Representative photomicrographs of tubular structures. Images were quantified by (**B**) counting the number of branch points and (**C**) calculating total tube length in low power fields. Data are shown as mean ± SEM of counted tube branch points and total tube length from six microscopic fields of three independent experiments. * *p* < 0.05 vs. *Malat1* Ctrl group. Scale bar represents 100 µm.

To further define the molecular mechanisms of *Malat1*-mediated angiogenic activity after ischemic injury, we next analyzed the potential effect of *Malat1* silencing on angiogenic activity in vitro by tube formation assays. Tube formation assay is known to mimic multiple key steps during angiogenic process, including endothelial cell adhesion, migration, differentiation, and growth [35]. *Malat1* GapmeR treatment markedly attenuated angiogenic tube formation of SMMECs compared with *Malat1* control group (Figure 6A), as quantitatively evaluated by number of branch points (Figure 6B) and total tube length (Figure 6C). Taken together, these results suggest that *Malat1* directly regulates angiogenic activity in mouse SMMECs.

### 2.6. Malat1 Physically Binds to VEGFR2

**Figure 7:**
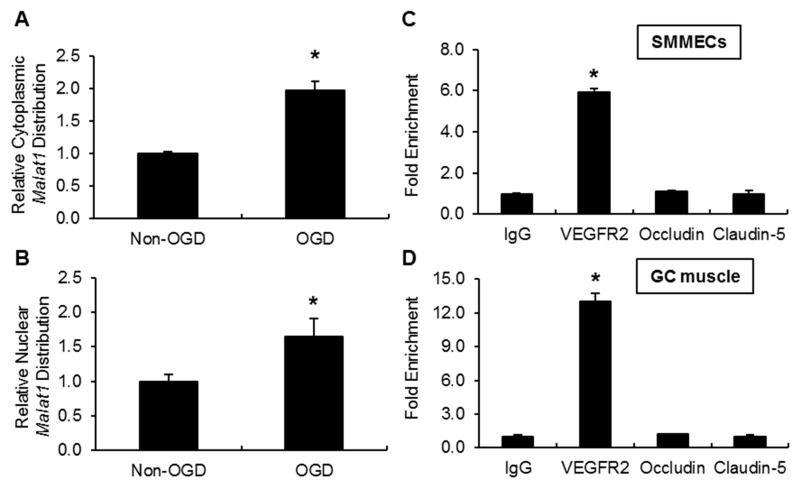
*Malat1* physically associates with VEGFR2 in mouse-cultured SMMECs and mouse gastrocnemius muscles. (**A**,**B**) The subcellular localization of *Malat1* was detected in mouse SMMEC cells. Nuclear, cytoplasmic, and total RNA were isolated from SMMEC cells with or without 16 h OGD treatment using the RNA Subcellular Isolation Kit (Active Motif, Carlsbad, CA, USA). Isolated RNAs were subjected to reverse transcription and qPCR analysis, with *Malat1* from total RNA as endogenous control. (**C**,**D**) Interaction between *Malat1* and VEGFR2 was revealed by RNA Immunoprecipitation. Total extracts of (**C**) cultured SMMECs and (**D**) mouse gastrocnemius muscles were immunoprecipitated with control IgG or anti-VEGFR2 antibody, and the complexes were analyzed for the presence of *Malat1* by qPCR. Relative gene expression levels were calculated using the formula 2^−Δ*C*t^ with Δ*C*_t_ = *C*_t_(target)__–*C*_t_(control)__ (*n* = 3). Data are expressed as mean ± SEM of two independent experiments with triplicate wells. * *p* < 0.05 vs. IgG group.

LncRNAs are known to exert biological effects by modulating gene expression and serve regulatory functions through sequence-specific hybridization and/or through structural and spatial mechanisms [36,37]. *Malat1* predominantly localizes to nucleus under physiological condition [38,39,40,41]. Here we determined the subcellular distribution of *Malat1* in mouse SMMECs by separating nuclear and cytoplasmic RNA fractions using a commercial kit. When we profiled *Malat1* distribution in each fraction after 16h OGD exposure, we observed ~2-fold enrichment of *Malat1* in the cytoplasm (Figure 7A) and ~1.5-fold enrichment of *Malat1* in the nuclear (Figure 7B) compared with the non-OGD group. One of the most straightforward explanations for *Malat1*’s regulatory effects on pro-angiogenic VEGFR2 is their physical association. Therefore, we performed RNA-immunoprecipitation (RIP) with VEGFR2 antibody from total extracts of OGD-treated mouse SMMEC cultures. We observed ~6-fold enrichment of *Malat1* in the VEGFR2 antibody immunoprecipitated SMMEC extracts compared with IgG control (Figure 7C). We also included two tight junction proteins claudin-5, and occludin in the RIP experiment, to serve as controls, as they have no interaction nor regulatory effects with *Malat1*. To confirm the physical interaction of *Malat1* with VEGFR2 in vivo, we performed RIP with VEGFR2 antibody from fresh gastrocnemius muscle extracts of C57BL/6J mouse. Consistently, we observed ~12-fold enrichment of *Malat1* in the VEGFR2 antibody immunoprecipitated extracts from gastrocnemius muscles compared with IgG control (Figure 7D). Taken together, the results indicate that *Malat1* is physically associated with VEGFR2 in vitro and in vivo.

## 3. Discussion

In this study, we addressed the potential role and molecular mechanisms of lncRNA *Malat1* in regulating cell-autonomous angiogenesis. We demonstrated that genetic deficiency of *Malat1* led to a significantly lower recovery of local blood flow and decreased capillary density in mice following hindlimb ischemia. Moreover, LNA-GapmeR mediated silencing of *Malat1* in skeletal muscle microvascular endothelial cells effectively reduced endothelial cell tube formation, migration, and proliferation. We further identified *VEGFR2* as a direct downstream target of *Malat1* through physical binding. The interaction between *Malat1* and *VEGFR2* is responsible for *Malat1*-mediated angiogenic effects after ischemic insults.

*Malat1* was one of the first identified lncRNAs associated with human disease, which was originally described to be associated with metastasis of lung cancer [42]. It has been reported that *Malat1* drives tumorigenesis through the promotion of tumor cell proliferation [43]. Moreover, overexpression of *Malat1* results in increased cell migration in vitro [43]. Depletion of *Malat1*, on the other hand, inhibits cell motility in vitro and significantly limits metastasis formation in mouse cancer models [43,44]. In addition, *Malat1* was reported to play an important role in tumor-driven angiogenesis through regulating vasculature formation and fibroblast growth factor 2 expression [34]. It has also been reported that *Malat1* is involved in diabetes-induced microvascular dysfunction and regulates retinal endothelial cell proliferation, migration, and tube formation [29]. In another study, genetic deletion of *Malat1* demonstrated reduced retinal vascular growth and endothelial growth [32]. However, the direct role of *Malat1* on vascular endothelial cells in the setting of angiogenesis (cell-autonomous angiogenesis) has not been clearly identified despite its extensive expression in the vasculature.

In our in vivo experiments, genetic deficiency of *Malat1* appeared to significantly attenuate ischemia-induced angiogenesis, perfusion, and functional recovery of ischemic hindlimbs by impairing the expression of VEGFR2. In vitro studies using mouse primary skeletal muscle microvascular endothelial cells found that LNA-GapmeRs mediated *Malat1* silencing resulted in significantly reduced EC tube formation, cell migration, and proliferation. Interestingly, although our in vivo results were in agreement with observations made by the Michalik group, i.e., that pharmacological inhibition of *Malat1* significantly inhibited blood flow recovery in a mouse hindlimb ischemia model [32], our in vitro observations were slightly different. Both siRNA- and GapmeR-mediated silencing of *Malat1* induced angiogenic sprouting but repressed proliferation of HUVEC cells [32]. On the other hand, the Liu group reported that siRNA-mediated *Malat1* silencing significantly reduced vasculature formation capacity of HUVEC cells under both normoxic and hypoxic conditions [34]. In another study, siRNA-mediated silencing of *Malat1*-inhibited retinal endothelial cell migration and tube formation [29]. The discrepancy between two opposite in vitro observations could be due to different endothelial cell lines chosen for each in vitro study. Nonetheless, our results indicate that *Malat1* silencing inhibited angiogenesis both in vivo and in vitro.

LncRNAs are predominantly located in the nucleus, and few are located in the cytoplasm [45]. *Malat1* has been known as a nuclear-retained non-coding RNA [46]. The Michalik group determined the localization of several lncRNAs, including *Malat1*, in endothelial cells derived from various human vascular beds and found that *Malat1* was highly enriched in the nuclear fraction [32]. The subcellular distribution of *Malat1* in BMECs was determined in our previous study by in situ hybridization [30]. It appeared that *Malat1* was predominantly localized in the nucleus, which is consistent with most other published studies in HeLa cells [47], lung cancer cells [44], and THP-1 cells [39]. However, several recent publications reported the presence of *Malat1* in the cytoplasm to exert its function [48,49]. The Yang group profiled *Malat1* distribution throughout the cell cycle [49]. Interestingly, they observed that *Malat1* was partially translocated from the nucleus to the cytoplasm in the G2/M phase in synchronized HeLa cells, which contrasted to the unsynchronized cell populations in which *Malat1* was localized predominantly in the nuclear portion. In our study, the subcellular distribution of *Malat1* was determined using mouse SMMECs and the separation of nuclear and cytoplasmic fractions were made using a commercial kit. We observed ~2-fold increased *Malat1* expression in cytoplasmic fraction after 16 h OGD exposure when compared with *Malat1* level in cytoplasmic fraction without OGD treatment, indicating that *Malat1* subcellular distribution may be cell type-specific and varies under physiological and pathophysiological conditions.

The most straightforward explanation for *Malat1* regulation of VEGFR2 is through physical interaction. Previous work by the Zhao group utilized RIP technique with the p65 antibody from subcellular extracts of LPS-stimulated THP-1 macrophages and observed ~7-fold enrichment of *Malat1* in the anti-p65 immunoprecipitated cell extracts compared with the IgG control [39]. We also employed an RIP experiment in our study, and we observed ~6-fold enrichment of *Malat1* in the anti-VEGFR2 immunoprecipitated extracts of OGD-treated SMMEC cultures and ~20-fold enrichment of *Malat1* in the anti-VEGFR2 immunoprecipitated gastrocnemius muscle extracts compared with IgG controls. This was the first study to document a direct physical association between *Malat1* and *VEGFR2* in vitro and in vivo.

The endothelium forms the central vascular barrier to maintain physiological vessel function and integrity. The angiogenic function of endothelial cells is strictly regulated by complex mechanisms, but the impact of lncRNAs has been less studied. Angiogenic response can be triggered under ischemic conditions such as ischemic stroke and myocardial infarction [6,50]. Post-ischemic angiogenesis plays a crucial role in the recovery of blood flow in ischemic tissues [51,52]. It has been reported that, in both stroke and heart attack patients, increased microvessel density has been observed in the penumbral areas, and the number of new angiogenic vessels is correlated with longer survival [53], suggesting active angiogenesis may be beneficial for neurological or cardiac functional recovery.

## 4. Materials and Methods

### 4.1. Experimental Mice

*Malat1* knockout (*Malat1* KO) mice on a C57BL/6J background were kindly provided by Dr. David L. Spector [28]. Generally, *Malat1* KO mice are viable and fertile with normal appearance, behavior, growth, and litter size [26]. Animal studies were designed and performed following University of Pittsburgh Institutional Animal Care and Use Committee approved protocol (15096911, 22 June 2017).

### 4.2. Mouse Model of Hindlimb Ischemia

Eight-week-old female *Malat1* KO and littermate control (*Malat1* WT) mice were anesthetized with 1.5–3% isoflurane (Henry Schein Animal Health, Melville, NY, USA). The right femoral artery was ligated and excised as described previously [20,54,55]. The mice were kept alive for four weeks to monitor local blood flow recovery and later subjected to histological analysis. A sham procedure was performed on the contralateral hindlimb to serve as the internal control.

### 4.3. Laser Speckle Imaging (LSI) Measurement of Focal Blood Flow

A laser speckle imager (Perimed PeriCam PSI HR, Stockholm, Sweden) was used to assess the recovery of local blood flow in mice after hindlimb ischemia procedure. Measurements were performed before surgery (Pre), immediately after surgery (D0), and postoperatively at days 7, 14, 21, and 28. The hair was removed from the ventral hind limbs of the mice before laser scanning to minimize data variations. After three consecutive scans over the same region of interest (hind legs and feet) in each animal, the average blood flow values of ischemic and non-ischemic limbs were calculated by computer-assisted quantification. The perfusion index was determined as the ratio of ischemic to non-ischemic hindlimb blood flow [20].

### 4.4. Analysis of Capillary Density

Capillary densities were examined by counting the number of capillaries stained with anti-CD31 antibody. Ten different random microscopic fields on five different sections from each animal were photographed and analyzed by Image J (National Institutes of Health, Bethesda, MD, USA). Capillary density was expressed as number of capillaries per high-power field (400×) as previously described [20].

### 4.5. Immunofluorescent Staining and Confocal Imaging

After completion of LSI, sham-operated and ischemic *Malat1* WT and KO mice (*n* = 5 per group) were perfused with saline, and the gastrocnemius muscles were dissected out from both ischemic and contralateral hindlimb. Coronal sections (8 µm) were cryosectioned for immunostaining with rat anti-CD31 (BD Pharmingen, San Diego, CA, USA 1:200) or rabbit anti-VEGFR2 antibody (Cell Signaling Technology, Danvers, MA, USA, 1:200). Secondary antibodies: Cy3-conjugated goat anti-rat IgG, Alexa Fluor 488-conjugated goat anti-rabbit IgG (all at 1:1000; Jackson ImmunoResearch Laboratories, Inc., West Grove, PA, USA). Images were collected on confocal microscope (FV1000-II; Olympus; Tokyo, Japan) and processed in Adobe Photoshop (CS2 version 9.0, Adobe Systems, San Jose, CA, USA) for compositions [56].

### 4.6. Cell Cultures and Oxygen-Glucose Deprivation

C57BL/6 mouse primary skeletal muscle microvascular endothelial cells (SMMEC) were purchased from Cell Biologics (C57-6220). Mouse SMMEC cells (2–6 passages) were grown to 85–95% confluency before use. To mimic ischemia-like conditions in vitro, mouse SMMEC cultures were exposed to OGD for 16 h. In some experiments, mouse SMMECs were treated with 50 nM *Malat1* GapmeR (5’–3’/56-FAM/CGTTAACTAGGCTTTA) or Negative Control A (*Malat1* Ctrl) (5’–3’/56-FAM/AACACGTCTATACGC) for 48 h prior to OGD exposure [26].

### 4.7. Capillary Tube Formation Assay

*Malat1* Ctrl/GapmeR-treated SMMEC cells were seeded on Matrigel-coated 24-well plates (1 × 10^5^ each well) and incubated for 6 h. Live cell images were collected using EVOS XL microscope (Waltham, MA, USA). Tube formation was analyzed by Image J and quantified by counting the number of branch points and calculating the total tube length in six randomly chosen fields from each well [20].

### 4.8. In Vitro Scratch Assay

SMMEC cells were transfected with either *Malat1* Ctrl or *Malat1* GapmeR for 48 h in 6-well plates. The SMMECs were scraped with a yellow pipet tip, and live cell images were collected using EVOS XL microscope at time 0 h and after 22 h incubation. Cell migration was calculated according to a published protocol [20,57].

### 4.9. Bromodeoxyuridine (BrdU) Cell Proliferation Assay

The effect of *Malat1* silencing on cell proliferation was determined by BrdU cell proliferation assay (Millipore, Burlington, MA, USA, 2750). Briefly, SMMEC cells were transfected with either *Malat1* control or *Malat1* GapmeR at 50 nM for 48 h and 72 h. The cells were then pulsed with BrdU reagents, and cell proliferation was measured by colorimetric immunoassays [20].

### 4.10. Quantitative Real Time

Total RNA was isolated from mouse SMMEC cultures or gastrocnemius muscle by using RNeasy Mini Kit (Qiagen, Valencia, CA, USA) or TRI reagent (Molecular Research Center, Inc., Cincinnati, OH, USA). Quantitative real-time reverse-transcriptase polymerase chain reaction (RT-PCR) was carried out with a Bio-Rad CFX Connect thermocycler, iScrip cDNA synthesis kit, and iTaq Universal SYBR green supermix (Bio-Rad, Hercules, CA, USA). Specific primers used for the reaction were as follows: *Malat1* Forward, 5’-ggcggaattgctggtagttt-3’; *Malat1* Reverse, 5’-agcatagcagtacacgcctt-3’. VEGFR2 Forward, 5’-tttggcaaatacaacccttcaga-3’; VEGFR2 Reverse, 5’-gcagaagatactgtcaccacc-3’. Cyclophilin Forward, 5’-actcctcatttagatgggcatca-3’; Cyclophilin Reverse, 5’-gagtatccgtacctccgcaaa-3’. The relative mRNA expression was normalized to Cyclophilin RNA levels. PCR experiments were repeated three times, each using separate sets of cultures [26].

### 4.11. Western Blot

Samples from the mouse SMMEC cultures or gastrocnemius muscle were homogenized in RIPA buffer (Thermo Scientific, Waltham, MA, USA), and total protein was isolated. Primary antibodies used were as follows: VEGFR2 (Cell Signaling Technology, Danvers, MA, USA, 1:1000) and β-actin (Sigma, Marlborough, MA, USA, 1:4000) [20,26].

### 4.12. RNA Subcellular Isolation

RNA subcellular isolation was performed using RNA Subcellular Isolation Kit (Active Motif, Carlsbad, CA, USA) according to the manufacturer’s instruction. Briefly, mouse SMMEC cultures with or without 16 h OGD exposure were lysed in complete lysis buffer. The lysate was then separated by centrifugation, with the supernatant containing cytoplasmic RNA and the pellet containing nuclear RNA. A guanidine-based buffer and ethanol were added to each RNA fraction before being loaded onto spin-columns. The RNA fractions were washed, eluted and quantified. Purified RNAs from each fraction were analyzed by qPCR using primers specific for *Malat1*. The relative cytoplasmic *Malat1* level was normalized to *Malat1* from total RNA extract.

### 4.13. RNA Immunoprecipitation

RNA immunoprecipitation (RIP) was performed using the Magna RIP RNA-Binding Protein Immunoprecipitation Kit (Millipore) as previously described [30]. Anti-VEGFR2 (Cell Signaling Technology) antibody was used for RIP. Co-precipitated RNAs from total extracts of mouse SMMEC cultures or mouse gastrocnemius muscle were analyzed by qPCR using primers specific for *Malat1*. Total RNA (input control) and the isotype control were assayed simultaneously to show the binding specificity between *Malat1* and VEGFR2.

### 4.14. Statistical Analysis

Quantitative data are expressed as mean ± SEM based on at least three independent experiments of triplicate samples. Differences among three or more groups were statistically analyzed by one-way analysis of variance followed by Bonferroni’s post-hoc test. Comparisons between two experimental groups were based on a two-tailed *t* test. A *p*-value less than 0.05 was considered significant.

## 5. Conclusions

Using genetically manipulated animal model and antisense LNA GapmeRs, our present findings suggest that *Malat1* regulates post-ischemic angiogenesis via interaction with *VEGFR2*. The findings will lead us to better understand the mechanisms of LncRNA-mediated regulation of angiogenesis and establish a human translational basis for the development of novel restorative therapy to enhance functional recovery following ischemic cerebrovascular and cardiovascular diseases.
